# Disability and Public Health

**DOI:** 10.3390/ijerph13010123

**Published:** 2016-01-12

**Authors:** Jerome E. Bickenbach, Alarcos Cieza, Carla Sabariego

**Affiliations:** 1 Swiss Paraplegic Research, Guido A. Zäch-Strasse 4, 6207 Nottwil, Switzerland; jerome.bickenbach@paraplegie.ch (J.E.B.); a.cieza@soton.ac.uk (A.C.); 2 Faculty of Social and Human Sciences, University of Southampton, Highfield Campus, Southampton SO17 1BJ, UK; 3 Department of Medical Informatics, Biometry and Epidemiology (IBE), Chair for Public Health and Health Services Research, Research Unit for Biopsychosocial Health, Ludwig-Maximilians-University Munich, Marchioninistr. 17, 81377 Munich, Germany

People with disabilities comprise approximately 15% of the world’s population. These are people with impairments, such as visual impairment and low back pain, and health conditions such as multiple sclerosis, spinal cord injury, depression and schizophrenia. This percentage is expected to increase, in part because of the world-wide ageing population and the steady increase in prevalence of chronic health conditions. More significantly, as authoritatively expressed in both the World Health Organization’s (WHO) International Classification of Functioning, Disability and Health [1], and the United Nations’ Convention on the Rights of Persons with Disabilities [2], disability is the outcome of the interaction between a person’s intrinsic state of health and their physical, human-built, attitudinal and social environment. More than ever, our scientific and political understanding of disability, and what it means to live with disabilities, depends on environmental research and, in particular, the environmental determinants of disability. 

This Special Issue brings together a variety of perspectives on the lived experience of disability and on the nature of the impact of the environment, broadly understood, in the context of public health. It is widely known that people with disabilities tend to experience lower levels of health due, not only to their primary and secondary health conditions and comorbidities, but also to the effects of social marginalization, poverty, denial of access to health and social services and discrimination. These crucial environmental factors either create disability or worsen disability. The time has come for public health to identify and directly respond to these environmental determinants.

Beyond planning, implementing and evaluating interventions, a core activity of public health is the epidemiological task of validly measuring disability across the population and tracking the health, as well as health and disability determinants, of populations. These are challenging tasks, because disability is a complex and multidimensional experience that creates substantial obstacles for description and measurement. Data about all dimensions of disability*—*information about impairments, restrictions of participation and the environmental factors that facilitate or hinder full participation*—*is essential to understand how disability plays out in people’s lives and to identify ways of socially responding to this experience. 

This Special Issue underscores the fact that disability is a central public health issue and one of increasing importance. The articles collected here address this salient fact from different perspectives and using different methodologies and study designs. There are four, interlocking and interconnected, themes represented in these articles:

## 1. Disability Measurement

One of the most persistent challenges in understanding the impact of the environment on disability is the basic epidemiological task of measuring disability phenomena across the population. Madden *et al*. report a substantial Australian effort to formalize an integrated measure that could be used to secure access to person-centred, policy-relevant information useful for a wide range of programs [3]. Failing to find an existing measure that was consistent with international trends in human services and public health, they set out to develop a generic, integrative measure of functioning for use in rehabilitation, disability support, and related fields. With regard to concerns about the structure of standard, national disability data collection strategies, Sabariego *et al*. [4] find that the standard method in censuses and population surveys of initially screening a population of ‘the disabled’ by means of basic health characteristics, tends to yield imprecise prevalence rates and classifies persons with mild to moderate disability as non-disabled, although they experience significant problems in daily life. They describe an alternative approach, recommended in WHO’s World Report on Disability [5], which uses a general population sample to directly measure the lived experience of disability in terms of restrictions of participation, and to define which persons are experiencing mild, moderate and severe disability using a metrical disability scale and cut-offs fit to purpose. This article attracted a response from Madans *et al*., [6] and Sabariego *et al*. [7].

## 2. Determinants of Health for Persons with Disabilities

McColl *et al*., in a Canadian study, address the important issue of access to primary health care, and in particular the kinds of obstacles that confront persons with disabilities when they try to access a family physician [8]. Smith and Caddick report the results of a qualitative UK study of individuals with spinal cord injury, discharged from rehabilitation into an elderly care home [9]. Their concern is with the quality of health care received in an environment characterized by a lack of freedom, control, flexibility and restricted participation in community activities. They report that the results are diminished physical health and psychological wellbeing in the short and long-term.

## 3. Disability and Public Health Interventions

Two articles in this Special Issue looked directly at public health interventions involving the environment that impacts on the experience of disability. Auger and colleagues in Montréal, Canada conducted an exploratory study to determine the usability of existing mobile applications for evaluating environmental barriers and facilitators, for persons with mild communication and cognitive limitations, when they experience a shopping mall [10]. The study recommends new features of these applications that might address unmet needs. In another Montréal study [11] Mazer *et al*. evaluated the structure, process and outcomes of a Community of Practice initiated and developed including stakeholders involved in a large multidisciplinary and multi-sectorial project: the Rehabilitation Living Lab in a Mall. The study strongly suggests that the Community of Practice approach to service delivery is a useful strategy to facilitate knowledge sharing on disability issues.

## 4. Environmental Impact on Specific Conditions

In the remaining three articles in this Special Issue, health and social service delivery approaches and other social determinants are evaluated in light of their impact on specific health conditions that are associated with disability. Brunani *et al*. present and evaluate an ICF-based individual rehabilitation project for obese patients with additional comorbidities [12]. They found that the service model they proposed not only standardizes rehabilitative procedures, simplifying organizational issues, but also facilitated the flow of useful data across episodes of care. Twomey *et al*. look at the impact of gender and general social-economic conditions on patterns of health service usage by older people with depression, suggesting that these environmental determinants, rather than severity of depression appear to account for the variance in usage [13]. Finally, Willems *et al*., consider the impact of environmental factors, in particular supportive relationships, on patients with disorders of consciousness [14]. Their results suggest that in order to prevent further decline in functioning patients with these disorders, they should be housed in a supportive and facilitative environment and caregivers should receive tailored support in order to enhance and facilitate appropriate care.
